# Risk factors for problematic smartphone use in children and adolescents: a review of existing literature

**DOI:** 10.1007/s40211-019-00319-8

**Published:** 2019-09-06

**Authors:** Linda Fischer-Grote, Oswald D. Kothgassner, Anna Felnhofer

**Affiliations:** 1 Department of Pediatrics and Adolescent Medicine, Medical University of Vienna, Waehringer Guertel 18–20, 1090 Vienna, Austria Austria; 2 Department of Child and Adolescence Psychiatry, Medical University of Vienna, Waehringer Guertel 18–20, 1090 Vienna, Austria

**Keywords:** Problematic smartphone use, Children, Adolescents, Smartphone addiction, Problematic Internet use, Problematischer Smartphone-Gebrauch, Kinder, Jugendliche, Smartphone-Abhängigkeit, Problematische Internetnutzung

## Abstract

**Background:**

The percentage of smartphone users—especially among minors—is growing, and so is the body of literature hinting at increasing rates of problematic smartphone use in children and adolescents. However, comprehensive reviews regarding this issue are still scarce.

**Objective:**

The main aim of this review was to provide an overview of studies focusing on specific risk factors predicting problematic smartphone use in children and adolescents.

**Methods:**

A literature search was conducted in Google Scholar and PubMed.

**Results:**

The search yielded 38 articles that met the criteria for inclusion in this review. Research regarding influencing factors such as gender, age, and social, family, and personality factors, as well as duration of use and use patterns, could be found. Results seem to cautiously suggest that using a smartphone for gaming and social networking might be risk factors, whereas having good friendships might constitute a protective factor. Also, female adolescents seem to be prone to a higher smartphone addiction risk than male adolescents. For family, school, and personality factors, results are still scarce, and more research is needed. Nevertheless, strict parenting, low self-control, and low self-esteem seem to increase risks for problematic use, whereas academic motivation and school success might decrease this risk.

**Conclusion:**

A concise theoretical conceptualization of problematic smartphone use and corresponding standardized measures are needed to increase comparability of future studies and to thereby add to a clearer understanding of this contested concept.

## Zusammenfassung

**Hintergrund:**

Der Prozentsatz von Smartphone-Nutzern – insbesondere Minderjährigen – steigt an, und ebenso wächst der Bestand an Forschung, der auf steigende Raten von problematischem Smartphone-Gebrauch bei Kindern und Jugendlichen hinweist. Dennoch gibt es bis jetzt wenige umfassende Übersichtsarbeiten zu diesem Thema.

**Ziel:**

Daher ist es das Ziel dieses Reviews, einen Überblick über jene Studien zu bieten, die sich mit spezifischen Risikofaktoren befassen, die problematischen Smartphone-Gebrauch bei Kindern und Jugendlichen vorhersagen.

**Methoden:**

Eine Literatursuche wurde in Google Scholar und PubMed durchgeführt.

**Ergebnisse:**

Die Suche ergab 38 Studien, die den Inklusionskriterien dieses Reviews entsprachen. Es ließ sich Forschung bezüglich Einflussfaktoren wie Gender, Alter, soziale und familiäre Faktoren, Persönlichkeitsfaktoren sowie Nutzungsdauer und Nutzungsmuster finden. Unter Vorbehalt scheinen Studienergebnisse darauf hinzuweisen, dass eine Nutzung des Smartphones für Gaming und soziale Medien Risikofaktoren darstellen, während gute Freundschaften einen Schutzfaktor bilden könnten. Ebenso scheinen weibliche Jugendliche anfällig für ein höheres Smartphone-Abhängigkeitsrisiko zu sein als männliche Jugendliche. In Bezug auf familiäre Faktoren, Schule und Persönlichkeitsfaktoren ist die Forschungslage noch spärlich und weitere Forschung wird benötigt. Dennoch scheinen strenge Erziehung durch die Eltern, geringe Selbstkontrolle und geringer Selbstwert das Risiko für problematischen Gebrauch zu erhöhen, während akademische Motivation und Schulerfolg dieses möglicherweise verringern.

**Schlussfolgerung:**

Es werden sowohl eine präzise, theoretische Konzeptualisierung des problematischen Smartphone-Gebrauchs als auch entsprechende standardisierte Messinstrumente benötigt, um die Vergleichbarkeit zukünftiger Studien zu erhöhen und so zu einem besseren Verständnis dieses umstrittenen Konzepts beizutragen.

## Introduction

In recent years, the worldwide percentage of smartphone owners and users has increased steadily [1]. With features including, among others, communication, Internet, and multimedia [2], smartphones—not least because of their ease of access [3]—have several advantages such as productivity enhancement, facilitated information seeking [3], and heightened pleasure via social interactions [4].

Despite these benefits, however, a growing body of literature hints at negative consequences and possible dangers associated with smartphones [3, 5, 6]. These include excessive use [3], increasingly uncontrollable behaviors such as constantly checking for notifications [4], mental health problems such as depression and anxiety [3, 7], and physical problems [8]. It has been argued that problematic smartphone use can be viewed as a form of behavioral addiction like gaming addiction or Internet addiction [2, 4, 9]. Symptoms commonly associated with behavioral addictions, such as tolerance, withdrawal, mood dysregulation, cravings, and loss of control, have also been found to be related to problematic smartphone use [10]. Based on these findings, and similar to Internet addiction, Demirci et al. [2] have suggested that smartphone addiction is characterized by an overuse of smartphones that interferes with the users’ daily functioning.

Nevertheless, smartphone addiction is included neither in the *Diagnostic and Statistical Manual of Mental Disorders, Fifth Edition* (DSM-5) [11] nor in the upcoming *International Classification of Diseases 11th Revision (ICD-11)* [12], although the DSM‑5 now lists diagnostic criteria for Internet gaming addiction with the need for further research [11], and the ICD-11 has included the diagnosis of (Internet) gaming disorder in its preliminary online version [12]. On the one hand, this is interpreted as an increasing awareness concerning the existence of smartphone addiction by some [9]. On the other hand, a recent review [6] concludes that, to date, evidence is not sufficient to support the existence of smartphone addiction.

Instead, the authors suggest the terms problematic or maladaptive smartphone use, which pertain to an excessive behavior with lower levels of impairment than addiction [6]. Excessive use is sometimes measured as duration of usage and usage frequency (e.g., see Bae [13]), and problematic use is considered an uncontrolled behavior leading to negative consequences in everyday life [14]. The terms problematic smartphone use and smartphone addiction seem to be used synonymously, based on the researchersʼ understanding of the underlying construct. Researchers who assume the observed behaviors meet addiction criteria seem to choose the term smartphone addiction (e.g., see Yen et al. [15]), whereas researchers who do not consider addiction criteria met choose to refer to problematic smartphone use [3, 6, 14].

Given this inconclusive terminology, synthesizing existing research regarding problematic smartphone use in children and adolescents is a challenge [16]. For instance, reported prevalence rates of children and adolescents with problematic smartphone use vary widely, from 5% [17] to about 50% [15]. This may be interpreted mainly as a result of the different operationalizations used in different studies. These include, among others, problematic phone use [15, 17], extensive use of mobile phones (e.g., see Sánchez-Martínez and Otero [18]), smartphone addiction risk (e.g., see Lee et al. [5] and Cha and Seo [9]), and smartphone addiction [19–21].

Similarly, research on risk factors for problematic smartphone use in children and adolescents has, to date, yielded inconclusive results. Overall, minors seem to be particularly vulnerable [5, 9], which could be related to difficulties in self-regulation [4] and immature control competencies [22]. Other factors possibly influencing maladaptive usage in children and adolescents include age [23–25], gender [10, 24, 25], social factors [24, 26], and personality [23, 25, 27, 28].

Despite the growing body of data, comprehensive reviews synthesizing key findings are still scarce. A meta-analysis [20] focuses only on India, and Park and Park [24] propose a model of smartphone addiction but without comparing different results and without considering age groups other than early childhood. Furthermore, as smartphone technology is advancing rapidly, new developments have arisen since 2014. Hence, a review focusing solely on mobile phone addiction seems to fall short. Therefore, this review sets out to provide an overview of studies on problematic or addictive smartphone use in children and adolescents, with the focus particularly on factors that increase the risk of problematic smartphone use.

## Methods

### Search strategy

**Fig. 1:**
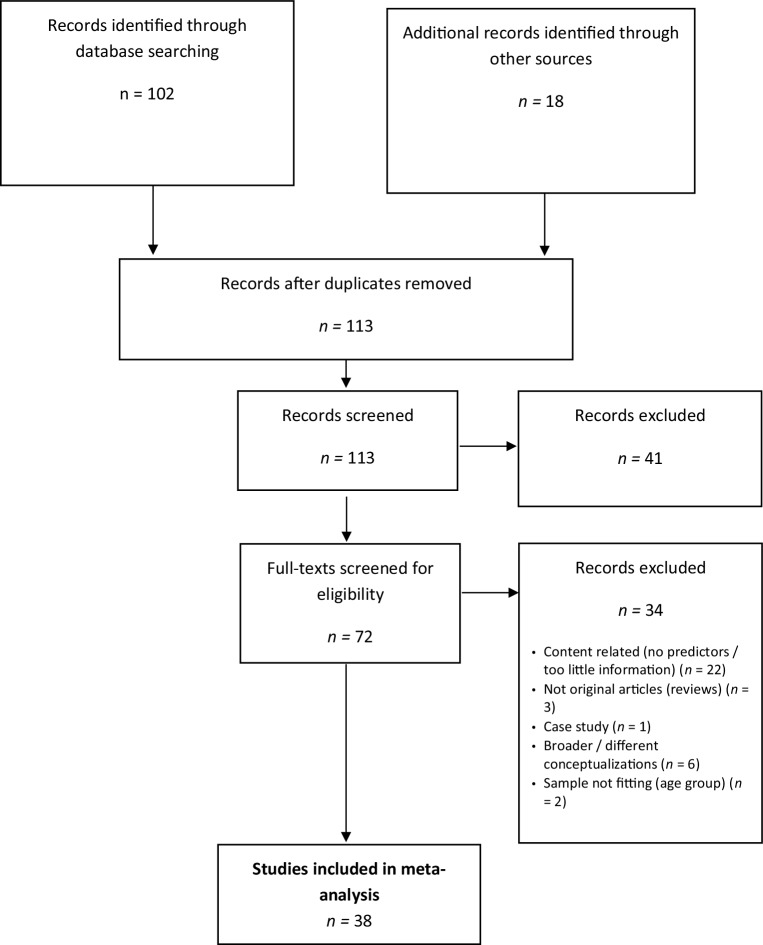
PRISMA flow diagram of the literature search

In order to increase the likelihood of including studies that focus on smartphones instead of older kinds of mobile phones (without Internet access), only publications from 2008 onward were searched, as done by Elhai and colleagues [3] in their review on problematic smartphone use and anxiety and depression. A literature search was conducted in Google Scholar and PubMed regarding papers published between January 2008 and May 2019. Search parameters were PROBLEMATIC/MALADAPTIVE/EXCESSIVE/PATHOLOGICAL/DYSFUNCTIONAL in combination with PHONE/SMARTPHONE/SMART PHONE/CELLPHONE/CELL PHONE/MOBILE PHONE and ADDICTION/USE as well as ADOLESCENTS/CHILDREN/YOUTH. Google Scholar alerts were enabled to ensure the inclusion of accepted articles and articles in preprint status. The title, abstract, and main text of each study were examined independently by the authors, and exclusions of studies occurred at each stage of the process (see Fig. 1). Additionally, a reference search strategy was used to identify other relevant articles.

### Study selection process

Articles were included if they were original articles, written in English, published in peer-reviewed journals not earlier than 2008, and focused primarily on children (1–10 years) and adolescents (11–21 years). Although the search focused on smartphones, publications regarding problematic cell phone or mobile phone use were also included, as it can be assumed that cell/mobile phones were used from 2008 onward at least by some participants. Articles focusing on positive effects of smartphones as well as editorials were excluded. Titles and abstracts found in the search were screened for relevance before full-text articles were reviewed. Studies examining college students were also included if a clear distinction between age groups was possible. Furthermore, risk factors were defined as variables that predict problematic smartphone use/smartphone addiction.

## Results

### Sample of included studies

The initial search yielded 120 articles. Eighty-two articles were subsequently excluded because they did not or did not exclusively examine children/adolescents, or because they were not original articles, were not peer-reviewed, were not written in English, or only examined Internet addiction or media use in general. Articles focusing on consequences of smartphone addiction were also not considered. For a detailed description of the exclusion process, see Fig. 1.

The final sample consisted of 38 articles. Of the included studies, 42.1% (*n* = 16) were conducted in South Korea and 13.2% (*n* = 5) in Turkey. Other studies were from Taiwan, China, Switzerland, Italy (each of these accounting for 5.3%), Germany, India, Poland, and Romania (each of these accounting for 2.6%). Two studies (5.3%) were conducted in Spain and one (2.6%) in the UK, and two additional studies (5.3%) were conducted both in Spain and the UK.

The Smartphone Addiction Scale–Short Version (SAS–SV) [10] or the longer version of the SAS [29] was used in 26.3% (*n* = 10) of the studies, and the Smartphone Addiction Proneness Scale (SAPS) [30] was used in 21.1% (*n* = 8) of the included studies. Of these, one study [31] applied both. The Mobile Phone Problem Use Scale for Adolescents (MPPUSA) [32] was used in three studies (7.9%), a shorter version of the Mobile Phone Problem Use Scale (MPPUS) was used in one study (2.6%) [16], and two studies (5.6%) applied a modified version of the Internet Addiction Scale by Young [33]. The remaining 14 studies each used a different measure (see Table 1).

**Table 1:** Study characteristics and results regarding risk factors of problematic smartphone use

Study	Sample size	Age	Gender	Country	Measure	Main results
Ayar et al. (2017) [34]	*N* = 609	M = 12.3 SD = 0.9	Female = 47.7% Male = 52.3%	Turkey	SAS V1	No effect of sociodemographic variables (age, parents’ educational level, monthly income levels) on smartphone addiction was found
Bae (2015) [35]	*N* *=* 2376 *N* = 2264 *N* = 2218	Primary school students (4th grade)	1. Female = 47.8% Male = 52.2% 2. Female = 47.9% Male = 52.1% 3. Female = 47.4% Male = 52.6%	South Korea	AUSS	More democratic parenting style was associated with less addictive smartphone use
						Increase in academic motivation was related to decrease in addictive smartphone use
						Increase in friendship satisfaction was related to decrease in addictive smartphone use
Bae (2017) [13]	*N* = 2212	13–18 years	Female = 48.6% Male = 51.4%	South Korea	S Scale	Frequency of smartphone use on weekdays and weekends was related to dependence
						Duration of use for information seeking, entertainment seeking, and gaming was related to dependence
						Duration of use for SNS and instant messenger was not related to dependence
Cha and Seo (2018) [9]	*N* = 1824	M = 15.6 SD = 0.78	Female = 49.0% Male = 51.0%	South Korea	SAPS	30.9% of participants were classified as a risk group for smartphone addiction
						Significant differences were found between addiction risk group and normal users regarding smartphone use duration, awareness of game overuse, and purposes of game playing
						Predictive factors: daily smartphone and SNS use duration, awareness of game overuse
Chóliz (2012) [36]	*N* = 2486	12–18 years	Female = 51.4% Male = 48.6%	Spain	TMD	Girls relied to a higher extent on the mobile phone; there were more negative consequences for girls
						Associations were found between TMD and use patterns
Cocoradă et al. (2018) [27]	*N* = 717	M = 19.8 (40% high school students)	Female = 65.0% Male = 35.0%	Romania	SAS–SV	High school students showed higher levels of addiction
						Girls showed higher levels of addiction
						Boys used more technology and for different activities
						High school students used smartphones more often and more for video gaming, phone calls, and TV viewing
						Correlations between personality traits, attitudes, and addiction were found
						Negative correlations existed between addiction and neuroticism, conscientiousness, and openness
De Pasquale et al. (2015) [28]	*N* = 200	14–19 years	Female = 42.0% Male = 58.0%	Italy	SAS–SV	Smartphone addiction was found only in boys, not in girls
Emirtekin et al. (2019) [37]	*N* = 443	M = 16.0 SD = 1.1	Female = 60.0% Male = 40.0%	Turkey	SAS–SV	Significantly higher score of problematic use was found in girls
						Emotionally traumatic experiences were associated with problematic use, partially mediated by psychosocial risk factors
Firat and Gül (2018) [38]	*N* = 150	M = 15.3 SD = 1.7	Female = 58.7% Male = 41.3%	Turkey	PMPUS	Higher level of problematic use was found in older adolescents
						Somatization, interpersonal sensitivity, and hostility predicted the risk of problematic smartphone use
Foerster et al. (2015) [16]	*N* = 412	12–17 years	Female = 61.4% Male = 38.6%	Switzerland	MPPUS-10	A higher score correlated with more time spent online and more online data traffic
Gallimberti et al. (2016) [39]	*N* = 1156	M = 12.0 SD = 1.0	Female = 46.5% Male = 53.5%	Italy	SMS–PUDQ	A positive association between problematic cellular phone use and having a larger circle of friends was found
Güzeller and Cosguner (2012) [40]	*N* = 950	1. M = 16.1 SD = 0.9 2. M = 16.0 SD = 0.9	1. Female = 56.0% Male = 44.0% 2. Female = 60.1% Male = 39.9%	Turkey	PMPUS	A correlation between problematic use and loneliness was found
Ha et al. (2008) [41]	*N* = 595	M = 15.9 SD = 0.8	Female = 7.2% Male = 92.8%	South Korea	ECPUS	Lower self-esteem was related to excessive mobile phone use
Haug et al. (2015) [42]	*N* = 1519	M = 18.2 SD = 3.6	Female = 51.8% Male = 48.2%	Switzerland	SAS–SV	Addiction was more prevalent in younger (15–16 years) than in older (>19 years) adolescents
Ihm (2018) [26]	*N* = 2000	M = 12.3 SD = 2.6	Female = 50.5% Male = 49.5%	South Korea	Adapted version of GPIUS 2	Social network variables were negatively related to smartphone addiction
						Higher level of addiction was associated with less social engagement
Jeong et al. (2016) [43]	*N* = 944	Sixth grade	Female = 49.0% Male = 51.0%	South Korea	Modified version of IAT	Children with lower self-control were more likely to be addicted to smartphones
						Those who used smartphones for SNS, games, and entertainment were more likely to be addicted
						Those who used smartphones for study-related purposes were not addicted
						SNS was a stronger predictor of smartphone addiction than gaming
						Sensation seeking and loneliness were not significant predictors
Kim et al. (2018) [44]	*N* = 3380	10–19 years	Female = 48.7% Male = 51.3%	South Korea	SAPS	Family dysfunction (domestic violence, parental addiction) was significantly associated with smartphone addiction
						Self-control and friendship quality were protective factors
Kwak et al. (2018) [45]	*N* = 1170	Middle school students	Female = 58.4% Male = 41.6%	South Korea	Modified version of IAT	Parental neglect was significantly associated with smartphone addiction
						Relational maladjustment with peers negatively influenced smartphone addiction
						Relational maladjustment with teachers had a partial mediating effect between parental neglect and smartphone addiction
Kwon et al. (2013) [10]	*N* = 540	M = 14.5 SD = 0.5	Female = 36.5% Male = 63.5%	South Korea	SAS–SV	Significantly higher scores existed in girls
Lee et al. (2016) [46]	*N* = 3000	13–18 years	Female = 47.3% Male = 52.7%	South Korea	SAPS	Frequent use of social networking site applications (apps), game apps, and video apps tended to exacerbate addiction to smartphones
						Active parental mediation was effective in young adolescent girls, technical restrictions were effective in young adolescent boys, and limited service plans were effective for both
						Parental restriction tended to increase likelihood of addiction
Lee and Lee (2017) [47]	*N* = 3000	Grades 7–12	Female = 47.3% Male = 52.7%	South Korea	SAPS	35.6% classified as addicts
						Students with high academic performance showed lower addiction rates
						Higher proportion of addicted females
						Attachment to parents and satisfaction with school life might serve as protective factors
						Motive for smartphone to gain peer acceptance was the most significant factor related to smartphone addiction
Lee et al. (2017) [21]	*N* = 370	1. M = 13.1 SD = 0.8 2. M = 13.3 SD = 0.9	Female = 50.8% Male = 49.2%	South Korea	SAPS	Addiction group showed significantly higher scores in online chat
						Purpose of use: addiction group showed higher levels of use for habitual use, pleasure, communication, games, stress relief, ubiquitous trait, and desire not to be left out
						Females: use for learning, use for ubiquitous trait, preoccupation, and conflict were significantly correlated with smartphone addiction
						Females: use for ubiquitous trait, preoccupation, and conflict were predictors
						Use for learning was a protective factor
Lee and Ogbolu (2018) [48]	*N* = 208	10–12 years	Female = 52.4% Male = 47.6%	South Korea	SAPS	Gender: no predictor of addiction
						Age, depression, and parental control positively predicted smartphone addiction
Lee et al. (2016) [5]	*N* = 289	M = 13.1 SD = 0.8	Female = 50.9% Male = 49.1%	South Korea	SAPS	Significantly more females were in the high-risk group
						Use per day was significantly higher in the high-risk group
Lee (2016) [49]	*N* = 490	M = 14.0 SD = 0.9	Female = 0% Male = 100%	South Korea	SAS–SV	High-risk group showed significantly lower self-esteem and poorer quality of communication with parents
						Severity of smartphone addiction was negatively associated with self-esteem
Liu et al. (2016) [50]	*N* = 689	M = 18.2 SD = 3.6	Female = 6.2% Male = 93.8%	Taiwan	SPAI–SF	Smartphone gaming and frequent use were associated with addiction
Lopez-Fernandez et al. (2014) [51]	*N* = 1026	M = 13.5 SD = 1.5	Female = 45.0% Male = 55.0%	UK	MPPUSA	Prevalence of problematic use: 10%
						Typical problematic user: 10–14 years, studying at a public school, considered themselves to be experts in this technology
Lopez-Fernandez et al. (2015) [52]	*N* = 2228 MPPUSA–sample: *N* = 1438	MPPUSA–sample: M = 14.2 SD = 1.7	Female = 48.2% Male = 53.8%	Spain UK	MPPUSA	Estimated risk showed stronger relationships with gender, age, type of school, parents’ education
						Being a girl, being older, going to private school, having a parent with a university degree were possible predictors of excessive mobile phone use
Lopez-Fernandez (2015) [17]	*N* = 2356	M = 14.1 SD = 1.7	Female = 39.1% Male = 60.9%	UK (52%) Spain (48%)	MPPUSA	Prevalence of problematic use: 14.9% in Spain and 5.1% in UK
						Patterns of usage were similar between British and Spanish students
						No gender differences were found
Randler et al. (2016) [31]	1. *N* = 342 2. *N* = 208	1. M = 13.4 SD = 1.8 2. M = 17.1 SD = 4.3	1. Female = 48.5% Male = 51.5% 2. Female = 70.2% Male = 29.8%	Germany	1. SAPS 2. SAS–SV	Girls were more prone to become addicted
						Age did not predict addiction
Sánchez-Martínez and Otero (2009) [18]	*N* = 1328	13–20 years	Female = 53.7% Male = 46.3%	Spain	Questionnaire designed for this study	41.7% were extensive cell phone users
						Significant associations of extensive phone use were found with age, sex, cell phone dependence, demographic factors
Seo et al. (2016) [53]	*N* = 2159	Middle and high school students	Female = 50.3% Male = 49.8%	South Korea	Items selected from KCYPS	Mobile phone dependency increased relationships with friends in girls
Soni et al. (2017) [19]	*N* = 587	M = 16.2–16.8	Female = 42.1% Male = 57.9%	India	SAS	Addiction scores were higher in males than in females
Sun et al. (2019) [54]	*N* = 1041	M = 12.4 SD = 0.7	Female = 44.5% Male = 55.5%	China	SAS V2	Child neglect, psychological abuse, and emotion-focused coping were risk factors for smartphone addiction
						Emotional intelligence and coping style mediated the relationship between neglect/abuse and addiction
Wang et al. (2017) [55]	*N* = 768	M = 16.8 SD = 0.7	Female = 56.0% Male = 44.0%	China	SAS–SV	Students with better student–student relationships were less likely to be addicted
						Students with higher self-esteem were less likely to be addicted
						Self-esteem was a mediator between student–student relationships and smartphone addiction
						This was moderated by the need to belong
Warzecha and Pawlak (2017) [56]	*N* *=* 470	16–20 years	Female = 61.1% Male = 39.9%	Poland	KBUTK	Around 35% at risk for smartphone addiction; around 4% showed smartphone addiction
						Higher amount of smartphone addiction and risk for smartphone addiction in girls than in boys
Yang et al. (2010) [57]	*N* = 11,111	M = 14.6 SD = 1.7	Female = 50.3% Male = 49.7%	Taiwan	PCPU–Q	16.4% had problematic cell phone use, girls more likely than boys
						<15 years were more likely to show problematic phone use
Yildiz (2017) [58]	*N* = 262	M = 16.6 SD = 1.1	Female = 50.4% Male = 49.6%	Turkey	SAS–SV	External-dysfunctional emotion regulation, internal-dysfunctional emotion regulation, and internal-functional emotion regulation significantly predicted Internet and smartphone addiction
						Emotion-regulation strategies explained 19% of variance in smartphone addiction

*N* sample size, *M* mean, *SD* standard deviation, *SAS (V1)* Smartphone Addiction Scale – Version 1 ([59], cited by [34]), *SAS* Smartphone Addiction Scale – Original Version [29], *AUSS* Addictive Use of Smartphone Scale ([60], cited by [35]), *S Scale* scale to measure smartphone dependence from the Survey on Internet Overdependence ([61], cited by [13]), *SNS* social networking services, *SAPS* Smartphone Addiction Proneness Scale [30], *TMD* Test of Mobile Phone Dependence [36], *SAS–SV* Smartphone Addiction Scale—Short Version [10], *PMPUS* Problematic Mobile Phone Use Scale [62, 63], *MPPUS-10* Mobile Phone Problem Use Scale–Short Version [16], *SMS–PUDQ* Short Message Service (SMS) Problem Use Diagnostic Questionnaire [64], *ECPUS* Excessive Cellular Phone Use Survey [41], *GPIUS 2* Generalized Problematic Internet Use Scale 2 [65], *IAT* Internet Addiction Test [33], *SPAI–SF* Short-form Smartphone Addiction Inventory [66], *MPPUSA* Mobile Phone Problem Use Scale for Adolescents [32], *KCYPS* Korean Children and Youth Panel Survey [67], *KBUTK* Mobile Phone Addiction Assessment Questionnaire [68], *SAS (V2)* Smartphone Addiction Scale – Version 2 ([69], cited by [54]), *PCPU–Q* Problematic Cellular Phone Use Questionnaire [57]

Regarding gender, most studies (*n* = 35, 92.1%) had an almost equal distribution of male and female participants (50% ± 15%). One of the included studies examined only boys [49], and two other studies had a ratio of about 94% boys to 6% girls [50] and 93% boys to 7% girls [41], respectively.

### Risk factors

#### Gender

Several studies identified female gender as a risk factor [5, 21, 31, 36, 52, 56], reporting significant positive associations between female gender and problematic usage in adolescents (13–20 years) [10, 18, 37, 47]. Contrary to this, some studies reported smartphone addiction only in boys [28] or found higher scores in boys than in girls [19, 45]. Finally, no influence of gender was detected in other studies [17, 48, 50]. Additionally, one study [27] showed that boys and girls use their phones for different reasons: Girls spend more time on social media or text messaging, while boys are more interested in video gaming, media sharing, and Internet searches.

#### Age

Most studies found age to predict problematic usage [48, 52] or to be associated with it [18, 27, 38, 39, 51]. Yet, some found older adolescents [38, 48, 52] or older girls [39] to be at a higher risk, whereas others found a higher prevalence in younger (11–14 years) than in older pupils (15–18 years) [51] or in high school students than in university students [27, 42]. However, others found no predictive value of age [5, 31, 34].

#### Duration of use

A higher frequency of smartphone use [13, 50], a higher duration of daily usage [5, 9] (on average 33.17 min longer than healthy users, [9]), and a higher habitual use [21] have all been found to be related to problematic usage. Similarly, more time spent online and a higher amount of mobile data traffic [16] were found to be associated with addiction.

#### Use patterns

Using the smartphone for social networking services (SNS) [43, 46] and the duration of this usage [9] both seem to predict smartphone addiction. Adolescents with problematic use patterns also spent more time on SNS [5] or in online chats and used the smartphone more often for communication [21]. Another study [13], however, failed to find an association between smartphone addiction and SNS or instant messenger use.

In addition, gaming [21, 43, 46, 50] and a stronger denial of game overuse [9] were also found to predict smartphone addiction. Time spent gaming on the smartphone was shown to be positively related to addiction [13]. Furthermore, adolescents with problematic usage engaged in gaming more habitually and more often to achieve targets [9].

Another predictor of addiction seems to be entertainment [43]. Seeking pleasure [21] and entertainment via smartphones by watching videos [13, 46], listening to music [13], or reading e‑books [13] have all been found to be associated with problematic use. Furthermore, adolescents with problematic smartphone use have been shown to use the phone more for the purpose of stress relief or preoccupation, in cases of conflict [21], to gain peer acceptance [47], and to avoid being left out [21]. Finally, one study reported frequency of information seeking to be a risk factor for smartphone addiction [13].

#### School

On the one hand, a study identified going to a private school as a predictor for excessive mobile phone use [52]. On the other hand, higher school success [39, 47] and satisfaction with school life [47], as well as reading books [39] and an increase in academic motivation [35] seem to be negatively correlated with addiction rates.

#### Family factors

Sociodemographic variables including parental educational background and monthly income were found to have no effect on smartphone addiction in one study [34], yet another was able to show a significant positive association between family income and intensive phone use (as defined by the frequency of usage and monthly phone bills) [18]. Furthermore, parental punishment [21], as well as restrictive mediation by parents (e.g., restricting access to apps) [46, 48] all seem to increase the likelihood of problematic use and addiction in children and adolescents, whereas attachment to parents [47] and a democratic parenting style [35] seem to serve as protective factors. A significant effect has also been found for domestic violence, parental addiction (substance abuse or gambling problems) [44], parental neglect [44, 45, 54], psychological abuse [54], and emotionally traumatic experiences, the latter being partially mediated by body image dissatisfaction, social anxiety, and depression [37]. The association between parental neglect and smartphone addiction seems to be partially mediated by dysfunctional relationships with teachers [45], emotional intelligence, and coping styles [54].

#### Social network

A positive association has been found between problematic smartphone use and larger circles of friends [39]. In contrast, social network variables [26], friendship quality [44], friendship satisfaction [35], better relationships between students [55], and social engagement [26] may constitute protective factors. Finally, including loneliness as a risk factor for problematic smartphone usage produced inconclusive results: While one study [40] found a positive correlation, another did not detect a significant relationship [43].

#### Personality

The likelihood of being addicted to smartphones seems to be higher in adolescents with lower self-control [43, 44]. Furthermore, low self-esteem [41, 49, 55, 57] as well as depression [48], somatization, interpersonal sensitivity, and hostility [38] seem to be correlated with problematic phone use.

A study examining personality traits [27] found neuroticism, conscientiousness, and openness to be negatively correlated with smartphone addiction. Another study found a significant negative correlation between the Smartphone Addiction Score and emotional stability, but found no significant associations with extroversion, conscientiousness, agreeableness, or openness to experiences [28]. In addition, emotion-focused coping [54] and external-dysfunctional, internal-dysfunctional, and internal-functional emotion regulation strategies [58] have been found to explain smartphone addiction to some extent. Sensation seeking, however, does not seem to predict smartphone addiction [43].

## Discussion

Although research regarding problematic smartphone use in children and adolescents covers many potential risk factors, the results are somewhat conflicting. Several aspects might have contributed to these contradictory results.

First, constructs examined by different questionnaires were not the same. The most frequently applied measure [10, 27, 28, 31, 37, 42, 49, 55, 58] was the Smartphone Addiction Scale (SAS–SV [10]). With eight studies referencing it [5, 9, 21, 31, 44, 46–48], the Smartphone Addiction Proneness Scale (SAPS [30, 70]) was the second most frequently applied assessment. Both measures assess the construct of smartphone addiction. Other measures pertained to the operationalization “mobile phone use.” Additionally, unrelated measures were adapted by authors for the purposes of their studies (e.g., see Young [33, Kwak et al. 43, and Jeong et al. 45]). The use of such a wide range of questionnaires is merely a symptom of a field of research that has yet to define its key research subject. Hence, operationalizations between questionnaires differ, and in many cases the term “cell phone” is simply substituted with the term “smartphones” [30]. In sum, most studies refer to smartphone addiction, whereas the term “problematic use” seems to be prevalent particularly in studies referencing mobile phones or cell phones. These heterogeneous operationalizations may again be understood as a reflection of the lack of a clear and concise conceptualization of the phenomenon. Hence, the need to reach a mutually accepted comprehensive definition of problematic smartphone use is a conditio sine qua non for further progress in the field.

Second, while the time frame (2008–2019) for the search was deliberately chosen so that the probability of including papers that focus on smartphones was increased, it is possible that participants were indeed using mobile phones without Internet access. It is not always clear which type of phone is referenced in studies and which specific features these phones had. This may substantially bias the conclusions drawn here. For example, time spent online [16] and on SNS [5, 9, 21, 46] has been identified as a possible predictor for smartphone addiction, yet in phones without Internet access, problematic usage is automatically precluded. Upcoming studies should therefore pay attention to precisely describing the type of phones studied as well as their available and actually used functionalities.

Third, all the studies included in this review focused on adolescents or mixed samples, whereas studies on young children (aged 1–10 years) are considerably scarcer. This is due to a higher prevalence of use and ownership of smartphones in adolescents. Yet in the past years, smartphone usage rates have also considerably increased among preschool children aged 6–10 years (see [71]). Hence, future research should include younger samples, as well as make an effort to not only focus on the role of parents in mediating media use, as done by those studies including younger children (e.g., see Hwang et al. [72]), but to assess child experiences directly.

It may be noticed that most studies (42.1%) that met criteria for inclusion came from South Korea. In comparison to other nations, ownership of smartphones has been found to be highest in South Korea [73], and about 96% of adolescents from South Korea use a smartphone [9]. Furthermore, studies suggest that cultural factors such as individualism vs. collectivism may have a significant influence on technology usage and technology acceptance in general [74] as well as on Internet addiction in particular [75]. For instance, a study evaluating the underlying factor structure of the Internet Addiction Test (IAT) in three collectivistic and individualistic cultures (United States, China, Africa) found the psychometric constructs to differ significantly across cultural, economic, and technological contexts [75]. Translated to the context of problematic smartphone usage, it is likely that the instruments used to assess addiction levels may have also overestimated or underestimated addiction rates as they may not have been designed to appropriately capture culturally shaped behaviors associated with smartphone use (e.g., whether smartphones are used more for mood modification in one culture than another; see Chen and Nath [75]). A more careful consideration of cultural factors in future research is needed to add to a better understanding of the generalizability and validity of the construct of smartphone addiction across cultural contexts.

Among possible predictors of problematic smartphone use, most factors produced contradictory findings. While a longer duration of use seems to be quite clearly associated with higher addiction scores, research on risk factors such as gender, social networks, and patterns of use remains inconclusive. Regarding age, findings suggest positive as well as negative correlations with smartphone addiction. Some findings exist about the influence of the school environment, as well as of family and personality factors, but corresponding data are still scarce.

Regarding gender, more studies seem to support the conclusion that female adolescents are more prone to a higher smartphone addiction risk than male adolescents. This is in line with a study in adults by van Deursen et al. [4], who also found a higher risk for smartphone addiction in women than in men. The authors relate this to the finding that women experience more social stress than men and that this results in gender-specific use patterns. Correspondingly, it has been shown that girls use their smartphones to a higher extent for social reasons than boys do [5, 27, 43, 47]. Boys, in turn, seem to focus more on gaming and media data sharing [27].

These differing usage patterns again fuel the debate about the conceptual validity of the construct at hand. One is inclined to argue that smartphone addiction, as it is currently defined (e.g., see Cha and Seo [9] and Kim et al. [30]), might be too broadly conceptualized. Similar to the dispute on whether the phenomenon of “Internet addiction” exists (e.g., see Widyanto and Griffiths [76]), it is conceivable that smartphone users may not be addicted to the device itself but to the applications provided by it (e.g., SNS, online games, online pornography). Thus, in correspondence to the suggestion made by Griffiths [77], a distinction needs to be made between addictions *to* the smartphone and addictions *on* the smartphone. It may, thus, be more promising to focus on specific types of use (e.g., problematic gaming as proposed by the ICD-11 [12]) in future research and assess relevant indicators of behavioral addictions (e.g., salience, mood modification, tolerance, withdrawal, conflict, and relapse [76]) in relation to specific applications rather than merely considering the frequency or duration of using the technical device in general.

## Limitations and conclusion

One of the limitations of this review is that the causality of the relation described for the variables in question is not statistically firm across all studies. Most studies included here used correlational research and were cross-sectional. Yet in order to debunk the question of directionality (i.e., whether a postulated risk factor is a contributor or a consequence), longitudinal research is warranted.

Despite interpretational difficulties due to different operationalizations, this review was able to provide an overview of risk factors related to problematic smartphone use or smartphone addiction in children and adolescents. Based on this, the following implications for future research may be postulated: Most importantly, a concise definition of the construct with a standardized terminology and operationalization would enhance the comparability of findings. Developing a comprehensive theoretical framework for this construct is, however, closely related to the question of whether it constitutes a singular entity that is sufficiently distinct from other concepts such as problematic gaming, or whether it is merely a symptom of the latter or of the addiction to specific applications on the smartphone.
